# Malaria Transmission in Bissau, Guinea-Bissau between 1995 and 2012: Malaria Resurgence Did Not Negatively Affect Mortality

**DOI:** 10.1371/journal.pone.0101167

**Published:** 2014-07-01

**Authors:** Johan Ursing, Lars Rombo, Amabelia Rodrigues, Peter Aaby, Poul-Erik Kofoed

**Affiliations:** 1 Projecto de Saúde de Bandim, Indepth Network, Bissau, Guinea-Bissau; 2 Malaria Research Laboratory, Unit of Infectious Diseases, Department of Medicine, Karolinska University Hospital, Stockholm, Sweden; 3 Department of microbiology, Tumor and Cell Biology, Karolinska Institutet, Solna, Sweden; 4 Department of Infectious Diseases Mälarsjukhuset, Eskilstuna, Sweden; 5 Centre for Clinical Research, Sörmland county council, Eskilstuna, Sweden; 6 Department of Paediatrics, Kolding Hospital, Kolding, Denmark; Université Pierre et Marie Curie, France

## Abstract

**Introduction:**

As *Plasmodium falciparum* prevalence decreases in many parts of Sub-Saharan Africa, so does immunity resulting in larger at risk populations and increased risk of malaria resurgence. In Bissau, malaria prevalence decreased from ∼50% to 3% between 1995 and 2003. The epidemiological characteristics of *P. falciparum* malaria within Bandim health and demographic surveillance site (population ∼100000) between 1995 and 2012 are described.

**Methods and Findings:**

The population was determined by census. 3603 children aged <15 years that were enrolled in clinical trials at the Bandim health centre (1995–2012) were considered incident cases. The mean annual malaria incidence per thousand children in 1995–1997, 1999–2003, 2007, 2011, 2012 were as follows; age <5 years 22→29→4→9→3, age 5–9 years 15→28→4→33→12, age 10–14 years 9→15→1→45→19. There were 4 campaigns (2003–2010) to increase use of insecticide treated bed nets (ITN) amongst children <5 years. An efficacious high-dose chloroquine treatment regime was routinely used until artemisinin based combination therapy (ACT) was introduced in 2008. Long lasting insecticide treated bed nets (LLIN) were distributed in 2011. By 2012 there was 1 net per 2 people and 97% usage. All-cause mortality decreased from post-war peaks in 1999 until 2012 in all age groups and was not negatively affected by malaria resurgence.

**Conclusion:**

The cause of decreasing malaria incidence (1995–2007) was probably multifactorial and coincident with the use of an efficacious high-dose chloroquine treatment regime. Decreasing malaria prevalence created a susceptible group of older children in which malaria resurged, highlighting the need to include all age groups in malaria interventions. ACT did not hinder malaria resurgence. Mass distribution of LLINs probably curtailed malaria epidemics. All-cause mortality was not negatively affected by malaria resurgence.

## Introduction

Malaria still causes an estimated 0.65–1.2 million deaths annually and *Plasmodium falciparum* is the main cause. The World Health Organization recommends use of long lasting insecticide treated bed nets (LLIN), indoor residual spraying and treatment with artemisinin based drug combinations (ACT) to control malaria. Encouraging reports from several malaria endemic sub-Saharan countries have described decreasing *Plasmodium falciparum* indices coinciding with the scale up of vector control measures and effective treatment [1]–[7]. However, the decline started prior to the scale up in several settings and it is not clear to what degree the various interventions have contributed to the decrease of malaria [3].

An obvious risk that follows decreased malaria prevalence is the risk for resurgence of malaria in a population with less protective immunity. The longer time the prevalence remains low, the older the age group that can be affected as a result of young children growing up without acquiring protective immunity. In line with this, the average age of children with malaria increased and a larger proportion of children with cerebral malaria compared to infants with severe anaemia have been seen to occur concurrently with decreasing malaria incidence [1], [8]–[10]. Furthermore, the mean age of children with severe malaria, the proportion of children with cerebral malaria and case fatality rate have been shown to be higher in low transmission settings compared to high transmission settings [11]. In a worst case scenario, malaria resurgence that affects older children may thus increase malaria attributed mortality. As immunity in a population wanes slowly, long term surveillance is essential to monitor the effects of successful malaria control interventions [9]. Furthermore, it is necessary to identify methods by which to stop malaria resurgence.

We have conducted back to back prospective clinical trials collecting data on children aged <15 years with malaria verified by microscopy at the Bandim health centre in suburban Bissau, Guinea-Bissau, since 1994. The area is part of the Bandim Health and Demographic Surveillance System (HDSS) within which all-cause mortality is monitored. This report describes the incidence of children with uncomplicated malaria and all-cause mortality in different age groups for a period of 18 years. The study period includes introduction of impregnated bed-nets and ACT's as well as the effect of at least 10 years of declining of malaria prevalence followed by malaria resurgence.

## Materials and Methods

### Ethics Statement

Patients were included into the respective clinical studies after verbal (before 2012) or written (since 2012) informed consent from their caretaker. Verbal consent was obtained as literacy rates were low. A study nurse read standardized information to children and caretakers and answered questions. After approval, she signed the clinical records form to document that informed consent had been obtained. A second study nurse was present during the process. In addition to the above the caretaker has since 2012 been required to sign or leave a thumb print to document that informed consent had been given. The method was approved by the ethical review board in Bissau, Guinea-Bissau. Ethical approval for the separate studies was granted by the ethical review board in Bissau, Guinea-Bissau, the regional ethics committee in Stockholm, Sweden the central scientific ethics committee in Denmark. Studies conducted since 2001 were also registered at ClinicalTrials.gov. References are given in previously published reports [12]–[21]. In addition the regional ethics committee in Stockholm, Sweden approved the molecular analyses done as part of this study (2011/832-32/2).

### Study area and population

The Bandim Health Project HDSS was established in 1978 and covers an area of 16 km^2^ in suburban Bissau, the capital of Guinea-Bissau. There are areas with rice paddies and plenty of pigs and fowl. The Bandim Health Centre is located centrally within the HDSS. Two similar health centres and the national hospital are within ∼4 km. At the Bandim health centre, two nurses see paediatric patients in the morning and one nurse during afternoons and weekends. There is a laboratory manned by two trained technicians equipped with microscopes.

Malaria microscopy has been available for free throughout the study period. Antimalarial drugs have been provided free of charge as part of the studies from 1994 to 2008 and in 2012. During an effectiveness study (2010–2012) drugs were not provided for free. However, artemether-lumefantrine was provided at a reduced cost as it was subsidised by external funding and treatment of recurring parasitaemia was free of charge.

### Assessment of population and all-cause mortality

All houses are mapped and censuses are conducted every 2–3 years. Registration and follow-up of children <3 years of age are done during 3-monthly household visits. All pregnancies, births and deaths and in and out migration of children less than three years of age are registered during these visits. The mid-year population was derived from the HDSS database and included all individuals registered in the area at the 1^st^ June of the respective year. The last census was done in 2009 and population estimates thereafter were extrapolated based on the annual increase between 1995 and 2009. In 2009 the population of the HDSS was 84 900 in the whole study area and 39,300 in Bandim I and II, the districts from which the majority of patients come.

### Data collection

Eleven clinical trials have been conducted back to back since 1994 [12]–[21]. The same basic inclusion criteria have been used in all studies: Children aged <15 years, residing within the HDSS, with fever or a history of fever, with a parasite density ≥800 *P. falciparum*/µl, without signs or symptoms of severe malaria were included after informed consent. Only 6 months of data were available from 1994 and there was a civil war in 1998. Therefore, 1994 and 1998 were not included. Due to lack of funding, data was not collected in 2009. Since 2010 rapid diagnostic tests have often been available but all children with possible malaria have, as before, also been screened using microscopy. Giemsa stained thick and thin smears were made from finger prick blood to identify species and quantify asexual parasitaemia (per 200 white blood cells) using 1000× magnification and a sunlit microscope. A slide was considered negative after examination of 100 high power fields.

### Malaria control interventions

The national malaria control programme provided data about interventions and coverage. Campaigns to distribute insecticide treated bed-nets (ITN) in 2000, 2003 and 2006 were aimed at children <5 years and pregnant women. Impregnation campaigns (2004–2010) were aimed at children <5 years but all nets brought by mothers were impregnated. In November 2011, LLINs were distributed to the whole population. The National Meteorological Institute, located approximately 2 km from the Bandim Health Centre, provided meteorological data.

### Statistics

There were occasional gaps in recruitment of patients for periods of 1–2 weeks between the end and start of studies. For trend analyses, the number of children included between studies, were assessed as the mean number of children included during the same period of time (e.g. 1 week) before and after the gap. The monthly incidence of malaria was calculated by dividing the number of children attending the Bandim Health Centre by the population in Bandim I and II from where the majority of patients came. This is probably an underestimate of the true incidence as antimalarial treatment may be acquired at other places. However, we have no indication that attendance at Bandim Health Centre has changed over time. Changes in incidence over time should therefore be comparable. Quantile regression was used to assess trends of changing age. Malaria incidence and mortality over time were analysed by Poisson regression using the robust estimator of variance with year as continuous covariate. The earliest time point was used as baseline as specified in the results section.

## Results

### Numbers, sex and age of children with uncomplicated malaria

There were 3522 cases of uncomplicated malaria verified by microscopy between June 1995 and December 2012. The sex distribution was 51% boys and 49% girls. In addition 81 (2%) cases of uncomplicated malaria were estimated to have occurred between the studies. Data are presented in Table 1.

**Table 1:** Baseline characteristics of children aged <15 years attending Bandim Health Centre and diagnosed with uncomplicated malaria.

Year	1995	1996	1997	1999	2000	2001	2002	2003	2004	2005	2006	2007	2008	2010	2011	2012
**Number of children with ** ***P. falciparum*** ** infection** *	214	211	172	377	266	301	256	343	180	172	109	40	141	316	362	143
Male**	97	83	92	173	124	146	146	160	97	84	47	22	73	167	205	76
Female**	106	116	80	184	134	145	110	183	83	88	42	18	64	149	154	67
**Median age (years)**	5	4	4	5	5	5	5	6	5	5	5	5	7	9	10	10
**Age inter-quartile range (years)**	2–7	2–8	2–7	3–7	3–8	3–9	3–9	3–9	3–9	3–8	3–8	3–7	4–10	6–12	7–12	7–12
Annual Rainfall (mm)**			1319		1809	1755	1215	1980	1801	1382	1310	1085	1743	1839	1412	1956

*Number of children with *P. falciparum* infection includes 81 estimated cases. ** Data on sex was missing in 7 children. *** In 2009 the total rainfall was 1524 mm.

### Incidence of uncomplicated Malaria

**Figure 1:**
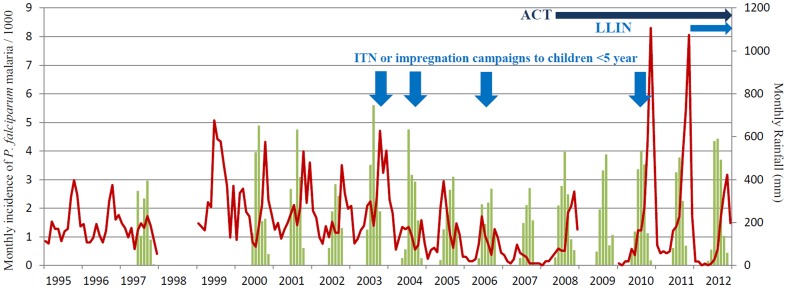
Monthly incidence of *P. falciparum* malaria per 1000 children aged less than 15 years attending the Bandim Health Centre and monthly rainfall (mm). Total monthly rainfall is shown as columns. Monthly *P. falciparum* incidence is shown as a line. Vertical arrows idicate when ITN or impregnation campaigns aimed at children <5 years of age were conducted. Horizontal arrows indicate the periods when artemisinin based combination therapy (ACT) and long lasting insecticide treated bed nets (LLIN) have been in use.

The monthly incidence of malaria, monthly rainfall, introduction of ACT and campaigns to increase the use of ITNs and LLINs are shown in Figure 1, Tables 2 and 3. Prior to the introduction of malaria specific interventions in Bissau, the mean annual incidence of malaria was 16 per 1000 (1995–1997). The incidence increased to 24 per 1000 in 1999–2003 (p<0.001) and subsequently decreased to 3 per 1000 in 2007 (p<0.001). Mean annual incidence then increased once again to 26 per 1000 by 2011 (p<0.001). Finally in 2012 malaria incidence again decreased to 10 per 1000 (p = 0.001 compared to 2011).

**Table 2:** Incidence of malaria and all-cause mortality in children aged less than 1, 1–4, 5–9 and 10–14 years per 1000 population.

	Year	1995	1996	1997	1999	2000	2001	2002	2003	2004	2005	2006	2007	2008	2010	2011	2012
	Age group																
***P. falciparum*** ** incidence**	**<1 year**	9	9	15	10	1	3	16	13	5	2	2	2	3	2	3	1
	**1–4 years**	27	28	24	48	33	35	29	31	20	18	10	4	10	12	10	4
	**<5years**	22	24	21	40	25	27	26	27	17	14	8	4	8	10	9	3
	**5–9 years**	19	13	13	34	26	28	19	35	15	16	9	4	13	30	33	12
	**10–14 years**	11	12	5	16	13	15	12	18	10	8	7	1	11	35	45	19
**All-cause mortality**	**<1 year**	116	124	126	154	108	89	97	136	99	97	77	79	83	73	78	39
	**1–4 years**	32	26	36	34	27	20	23	22	16	15	10	11	11	8	5	3
	**5–9 years**	5	7	6	8	4	3	5	4	3	3	2	2	3	2	0.2	0.4
	**10–14 years**	3	5	5	7	3	1	1	1	4	2	2	2	1	0.3	1	0

The mean annual *P. falciparum* incidence in all age groups was 16 per 1000 in the period 1995–1997 increasing to 24 per 1000 in 1999–2003 (p<0.001). Between 1995 and 1997 mean incidence was 22, 15, and 9 per 1000 in children aged <5, 5–9 and 10–15 years, respectively. Between 1999 and 2003 mean incidence for the same age groups was 29, 28, and 15 per 1000.

**Table 3:** Campaigns to treat bed nets and to distribute ITNs and LLINs in Bissau.

Year	Distribution of insecticide treated nets in the whole country	Number of nets treated as part of impregnation campaigns in Bissau	Estimated no of treated nets per person in Bissau
**2000**	10 000†		
**2003**	114 000†		
**2004**		15519	1/41
**2006**	181 925† *	33 892	1/11
**2010**		40 723	1/9
**2011**	880 000**		1/2

† Insecticide treated nets (ITN)

*Distributed in the whole country except Bissau. In Bissau vouchers were given for later collection of bednets but outcome of this is unknown.

** Long lasting insecticide treated nets (LLIN) were distributed in November

In addition there was an impregnation campaign in the whole country in 2005 but it is not known if this included Bissau.

**Figure 2:**
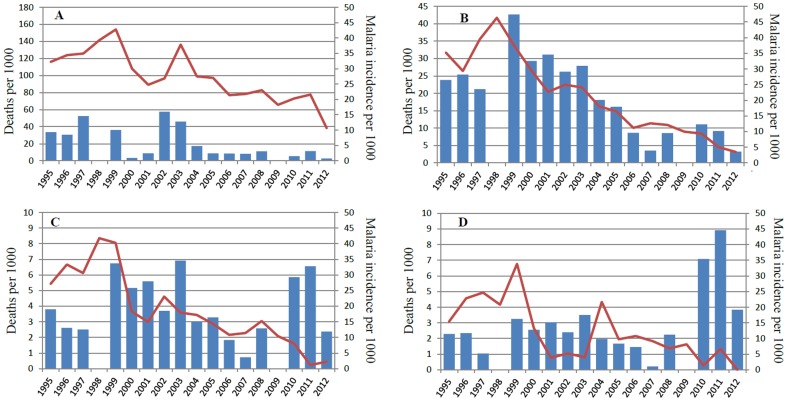
Annual malaria incidence and mortality per 1000 children aged less than 1 (A), 1–4 (B), 5–9 (C) and 10–14 years (D). Annual malaria incidence is shown as columns. Annual all-cause mortality is shown as a line.

The annual incidence of malaria in different age groups is shown in Table 2 and Figure 2.

Between 2003 and 2007 malaria incidence decreased 8, 7 and 15 fold (p<0.001) and then increased 2.5, 8.3 and 45 fold by 2011 in children aged <5, 5–9 and 10–14 years. Compared to

1999–2003 the incidence in 2011 was 1/3 in children aged <5 years but had increased 1.2 and 3 fold in children aged 5–9 and 10–14 years, respectively. By 2012 the incidence decreased 3.1, 2.6 and 2.1 fold compared to 2010–2011 in children aged <5, 5–9 and 10–14 years.

### Proportion of children aged <5, 5–9 and 10–14 years with uncomplicated malaria

**Figure 3:**
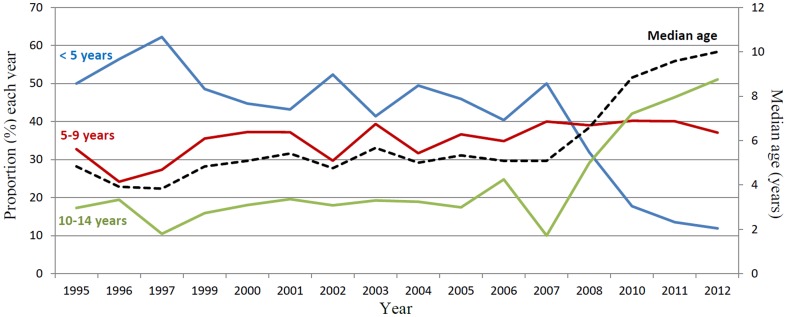
The annual proportion of children aged less than 5, 5–9 and 10–14 years with malaria and the median age of children aged less than 15 years attending the Bandim Health Centre and diagnosed with malaria.

The proportions of children aged <5, 5–9 and 10–14 years out of all children <15 years with malaria are shown in figure 3. The proportion of children <5 years decreased slightly between 1995 and 2003 as well as between 1995 and 2007. The proportion then rapidly decreased between 2007 and 2012 (p<0.001). The proportion of children aged 5–9 years increased gradually between 1995 and 2012 (p<0.001). The proportion of children aged 10–15 did not change between 1995 and 2007 but then rapidly increased between 2007 and 2012 (p<0.001).

### Age of children with malaria

The median ages of children with malaria each year are shown in table 1 and figure 3. Between 1995 and 2007 the median annual increase in age was 1.1 months (p<0.01); between 2007 and 2012 the median annual increase was 11 months (p<0.001).

### All-cause mortality

All-cause mortality and malaria incidence in children aged <1, 1–4, 5–9 and 10–14 years are shown in figure 2 and table 2. Between 1995 and peaks in 1998 or 1999 mortality increased significant in children aged 1–4 years and <5 years (p<0.01) and non-significantly in other age groups. There was a significant decrease in mortality in all age groups from the peak in 1998–1999 until 2012 (p<0.001).

### Interventions

The distribution of ITNs and LLINs and campaigns to impregnate bed-nets are shown in figure 1 and table 3. The effect of the 880 000 LLINs distributed in 2011 was assessed in a national survey of 1538 households (population of ∼16000) at the end of 2012. Compared with 2010, the number of households that owned an LLIN increased from 47% to 99%. In Bissau 84% (572/677) of distributed LLINs were used. Use of LLINs increased from 36% to 94% in children <5 years. The proportion of children <5 and the proportion of individuals of all ages sleeping under an LLIN the previous night were 97% (164/170) and 96% (1054/1099), respectively in 2012 [22].

An efficacious high dose chloroquine treatment regime was used until June 2008 when artemether-lumefantrine was introduced as national policy for treatment of uncomplicated malaria [18], [20], [23]. Quinine remained the drug of choice for severe malaria throughout. Chloroquine was also commonly used for home treatment of fever presumed to be malaria, at least until the introduction of artemether-lumefantrine. The changed treatment recommendation was widely announced by media and people were told to use artemether-lumefantrine and to stop using chloroquine.

During 2010 and 2011 artemether-lumefantrine was available at the Bandim health centre for 80/104 (77%) weeks. Rapid diagnostic tests were introduced in 2010 and were available for 60/104 weeks (58%). There has been no indoor residual spraying. The back to back clinical trials with follow up have ensured that all children with clinical malaria attending the Bandim health centre have received an efficacious treatment.

### Rainfall

Monthly and annual rainfall is shown in Figure 1 and Table 1. The mean annual rainfall during the study period was 1581 mm/year. Data was not available prior to 1997 and there were no records 1998–1999 due to the civil war. There was no trend of changing rainfall over the whole period. During the period of decreasing malaria cases 2003–2007, there was a parallel decrease in annual rainfall from a peak of 1809 mm in 2003 to a low of 1085 mm in 2007. Total annual rainfall increased after 2007 but fluctuated from year to year.

## Discussion

### P. falciparum incidence

The aim of this study was to describe variations in incidence of uncomplicated *P. falciparum* malaria and all-cause child mortality over time and in relation to malaria control measures. The small increase of malaria incidence 1995–2003 may in part have been a result of the civil war in 1998. However, concurrently with the increasing incidence, the *P. falciparum* prevalence decreased in Bissau. In suburbs of the capital Bissau including the Bandim HDSS, the prevalence was 26–59% in children aged <6 years in 1990 and 1993–1994 [24], [25]. By 2003/2004 the prevalence in children aged <5 years was only 3.6% and this remained unchanged at 3.2% in 2008 [26], [27]. As prevalence decreases it takes longer for children to acquire protective immunity. The population at risk thus becomes larger and older and more infections will be symptomatic. The first effects of decreasing malaria prevalence might thus paradoxically be increased incidence and increased age of children with symptomatic malaria [8]–[10]. In line with this hypothesis, the median age of children with malaria increased. This was primarily due to increased incidence and proportion of malaria in children aged 5–9 years between 1995 and 2003. Thus protective immunity had decreased in that age group in line with a gradual decrease of malaria prevalence since the mid 1990's.

The increased malaria incidence 1995–2003 was followed by an ∼8 fold decrease between 2003 and 2007. Similar decreases have been reported from several other African sites and have generally been attributed to ITNs, LLINs, indoor residual spraying and ACT [1]–[6], [28]. In Guinea-Bissau, including the Bandim HDSS, untreated bed-nets were widely used (79% in a survey in 2003/2004) and for that reason impregnation campaigns as well as distribution of ITNs were organised by the ministry of health between 2003 and 2010. Though the coverage was modest compared to the 2011 LLIN campaign, it is probable that part of the decrease was due to the impregnation campaigns [26]. The decrease of annual rainfall 2003–2007 probably also contributed.

As the prevalence of malaria decreases it has been suggested that malaria incidence will not change significantly (exempting an initial increase discussed above) due to an increased at risk population. This will apply until the prevalence reaches a threshold value. At that point the likelihood that an infection will be transmitted falls below one and malaria will decline rapidly [8], [9], [29]. Such a threshold was possibly reached in Bandim in 2003 when the prevalence in children aged <5 years was approximately 3.6%. This hypothesis is supported by the fact that ITN campaigns between 2003 and 2006 were mainly aimed at children <5 years whilst malaria decreased in all age groups. Had ITNs been the major driving force, one would expect malaria incidence to decrease selectively in children <5 years of age which was not the case. The rapid decline of uncomplicated malaria in Bissau between 2003 and 2007 was thus probably the end result of a long period of decreasing malaria prevalence prior to any intervention probably aided by ITNs and decreased rainfall.

The cause of the decreased malaria prevalence during the past 10–15 years is likely to be multi-factorial. A remarkable aspect is that the decline occurred despite the arrival of chloroquine resistant *P. falciparum* in Bissau in the late 1980's and the continued use of chloroquine [30]. A highly efficacious high-dose chloroquine treatment regimen was used in Guinea-Bissau until it was replaced by artemether-lumefantrine in June 2008 [20], [23], [30], [31]. Thus an efficacious drug was in use in Bandim as in the Gambia and Kenya where decreasing malaria indices were partly attributed to efficacious anti-malarials other than ACTs [1], [9].

The rapid increase of malaria incidence 2008–2011most probably represents a rebound of malaria in older children who lacked protective immunity. By 2003, the proportion of malaria primarily increased in children aged 5–9. When the epidemics started 5 years later, the age group 10–14 years was primarily affected. This suggests reduced malaria exposure since ∼15 years, i.e. since the mid-1990s, which tallies with experiences from other parts of Bissau [30]. In children aged <5 years malaria only increased ∼2 fold and in 2011 the incidence was 1/5 of that found in children aged 10–14 and 1/3 of the incidence prior to 2003. Thus the <5 year old age group was less exposed to malaria than in the past. ITN distribution and re-impregnation campaigns were primarily aimed at children aged <5 years suggesting that bed net use in Bissau accounted for some of the difference between age groups. A shift to outdoor biting mosquitoes that affect older children more is an additional possible explanation but we lack entomological data from the study area. The annual rainfall was higher in 2008, 2010 and 2011 compared to 2005–2007 and this may also have contributed to the increase of malaria. However, it does not account for the disproportionate increase of malaria amongst older children.

The disproportionate increase amongst older children may partly be due to an increased likelihood for older children to come to the health centre in order to obtain subsidised AL following the introduction of AL. However, this does not account for the gradual increase of malaria in children aged 5–9 years prior to the introduction of AL. There were also repeated stock-outs of AL and quinine was commonly prescribed, usually as injections that are more costly than unsubsidised AL. The incentive for changed health seeking behaviour may therefore have been small. Furthermore, assuming that younger children are as likely as older to seek help for presumed malaria, changed health seeking behaviour does not explain why the incidence of malaria was greater in children aged >10 years compared to children aged <5 years. The most plausible explanation is thus that the increased malaria incidence with age was a true reflection of malaria exposure and changes in immunity to malaria.

ACT has been suggested to decrease the prevalence of malaria in settings with good health care infrastructure due to its high efficacy and gametocytocidal effect [5]. Contrary to expectations, the number of children with clinical malaria started to increase soon after the introduction of artemether-lumefantrine in 2008 in Bissau. A randomised clinical trial that ended in 2008 found that the PCR corrected efficacies of artemether-lumefantrine and double dose chloroquine were ≥95% indicating that the resurgence of malaria was not due to poor efficacy. It is possible that changes in drug treatment policy interrupted efficacious informal intermittent presumptive treatment of fever with chloroquine (at home and at health centres). The change of treatment policy may thus inadvertently have enabled the epidemics accounting for the temporal association between the start of the malaria epidemics and introduction of ACTs.

Theoretically RDTs could have improved diagnostics to such an extent that the increase of malaria was simply a result of better diagnostics. However, RDTs were only available for 60/104 weeks (2010 and 2011). Furthermore, microscopy to verify *P. falciparum* infection has been done concurrently on all children with symptoms suggestive of malaria and only children with ≥800 *P. falciparum*/µl have been included in this study. It is therefore unlikely that RDTs have had a major impact on the number of children included in this study. Furthermore, in 2010–2011 approximately 40% of children had recurrent parasitaemia during the 42 day follow up. This was a far greater proportion than in previous studies. A large number of these children had re-infections (analysis is ongoing) but as they were already included in an effectiveness study they were only counted once. Thus the incidence of malaria was under estimated in 2010–2011 compared to previous years.

After 2 years of large scale epidemics the incidence of malaria again decreased in 2012. In November 2011, 880 000 LLIN were distributed in Guinea-Bissau and >95% of the population in Bissau of all age groups slept under an LLIN. Most probably, the cause of the reduction of malaria in all 3 age groups in 2012 was a direct consequence of this mass distribution of LLINs. Thus mass distribution of LLINs to all age groups can probably disrupt epidemics of malaria unlike limited campaigns aimed at specific age groups such as the 2010 re-impregnation campaign. Furthermore, preliminary data from 2013 indicate that malaria incidence has decreased even further. The incidence still increases with age though. The cause is beyond the scope of this paper but as mentioned above, an increase of exophilic mosquitoes might account for this. Several years of epidemics could have boosted immunity contributing to the decreased number of clinical cases in 2012. If immunity was a major factor causing the decreased incidence, one would expect less clinical malaria with increasing age. Seeing the opposite supports the idea that the decrease is primarily due to LLINs.

Our data comes from one health centre and can therefore not automatically be generalised. However, the decrease is supported by similar decreases between 2006 and 2007 at two other health centres in Bissau. Also, the prevalence of malaria was only 3.2% in <5 year olds and 1.3% in children and adults aged >5 years in a 2008 survey conducted in Bandim HDSS. Furthermore, in the same survey the prevalence in the whole country was ∼7% in individuals aged <5 and >5 years indicating a considerable decrease of malaria prevalence throughout Guinea-Bissau.

### All-cause Mortality

All-cause mortality decreased in all age groups during the study period. Decreasing malaria prevalence no doubt contributed to decreased mortality, especially as malaria has been seen to be linked to morbidity and mortality in other diseases affecting children [32]. The correlation between decreasing malaria incidence and decreasing all-cause mortality in children aged 1–4 years is particularly striking and similar to findings in Zanzibar following the introduction of ACT [5]. However, in contrast to Zanzibar, where only a small decrease of mortality was seen prior to the introduction of ACT, all-cause mortality and malaria incidence had been decreasing since 1999 in Guinea-Bissau. Though it is intriguing to note that an efficacious dose of chloroquine was routinely used in Guinea-Bissau there are no doubt multiple factors contributing to decreased malaria prevalence and mortality.

Another striking result was that there was no increase of all-cause mortality 2010–2011 despite malaria resurgence. In children aged <5 years, the incidence of malaria “only” doubled suggesting that protection from malaria (possibly by bed-nets) possibly contributed to the absence of increased mortality in this age group. However, in older children an up to 45 fold increase of uncomplicated malaria occurred representing a rebound of malaria. Malaria rebound in older children is a worst case scenario following successful reduction of malaria because of fears that this will result in increased malaria specific mortality due to a higher case fatality rate in older children [11], [33]–[35]. Our results indicate that rebound of malaria in a previously high endemic setting need not result in increased mortality. ACTs are rapidly acting and could conceivably reduce the risk of children developing severe malaria. However, preliminary data from the effectiveness study conducted 2010–2011 showed that quinine was preferentially given to children perceived to be more ill. Furthermore, 341/769 (44%) of children with uncomplicated malaria were prescribed quinine. It is thus unlikely that ACT explained the absence of malaria associated mortality

## Conclusion

We describe a decreasing incidence of uncomplicated malaria probably primarily attributed to a long period of decreasing malaria prevalence prior to major malaria control interventions. This was followed by resurgence of epidemic malaria over several years. Despite being introduced when malaria was at an all-time low ACT was not able to prevent malaria resurgence however, mass distribution of LLINs appears to have curtailed the malaria epidemics. Resurgence was probably possible due to a prolonged period with low malaria prevalence that created a large malaria susceptible population emphasizing the need for surveillance and early detection of increased malaria indices. Older age groups of children were particularly at risk of malaria during the resurgence highlighting the need to include all age groups in malaria control interventions. All-cause mortality decreased in parallel with decreasing malaria incidence but its role can-not be elucidated. An encouraging surprise was that rebound of malaria, including a 45 fold increase in the incidence of malaria in children aged 10–14 years, did not adversely affect all-cause mortality.
